# Counselling for Patients and Family Members: A Follow-Up Study in the Emergency Department

**DOI:** 10.5402/2012/303790

**Published:** 2012-09-12

**Authors:** Eija Paavilainen, Mari Salminen-Tuomaala, Päivi Leikkola

**Affiliations:** ^1^Nursing Science, Research Collegium, School of Health Sciences, University of Tampere, Etelä-Pohjanmaa Hospital District, 33014 Tampere, Finland; ^2^Nursing Science, School of Health Sciences, University of Tampere, Seinäjoki University of Applied Sciences, 33014 Tampere, Finland; ^3^Nursing Science, School of Health Sciences, University of Tampere, Etelä-Pohjanmaa Hospital District, 33014 Tampere, Finland

## Abstract

Although the research indicates that patients and family members are not fully satisfied with the counselling they receive, little is known about the quality of counselling in more detail. The purpose of the study was to describe patients' and their family members' experiences about counselling in emergency department, and follow how these experiences possibly change after the educational intervention for the whole nursing staff of the ED ward. The pre-test-post-test follow-up design was implemented including online continuing education for ED staff. The data were collected via questionnaires from patients and their family members in two phases and analyzed statistically. After online education of staff, experiences of patients and family members concerning counselling were better than before the education. Especially, family members' satisfaction had increased. However, our results also indicated that patients and family members desire more information for example, regarding medications. Care practices had developed towards family-centeredness, which patients and family members appreciate. Online education proved also in some degree its usefulness in educating ED staff, by offering the same education to a staff which works in shifts. Furthermore, family presence and participation practices should be developed by offering possibilities for families to stay with each other on ED ward.

## 1. Introduction

International health policy puts emphasis on counselling that enhances patients' health-related resources, and supports their independent coping abilities [1–3]. Counselling for patients discharged from the ED is important because they themselves and their family members are mostly responsible for care at home. The quality of counselling can be considered high if it meets the information needs and expectations of patients and their family members [4–8], helps them identify their resources and capabilities, and fosters their control over the situation [9]. 

During the past decade, the development of evidence-based nursing practice has been an internationally important issue [10]. Evidence-based practice serves the purpose of supporting nursing care that is based upon research, in a cost-efficient manner [11]. There is a need to develop the quality of care [12, 13] and as a part of it, family-centred counselling provided to patients and their family members in the ED [14, 15] as previous studies have indicated that patients and their families have not been fully satisfied with the counselling they received [16–18].

In order for patients to use their own resources and capabilities in an optimal manner, they need a sufficient amount of information about their illness and its treatment methods [18–21]. An insufficient amount of information on postdischarge care, such as medications and diet, and on how to recognize symptoms that can indicate that the patient's condition has worsened may lead to the failure of postdischarge care or the necessity to make a new appointment with a doctor, or even readmission [22]. When a patient is discharged, it would be important to map out his or her home circumstances, as patient coping may be impeded by health care needs that have gone unrecognized by the ED staff. Nurses' actions in an acute situation often tend to be technically oriented. It is important to enable patient and family participation in discharge planning [20, 23, 24] to ensure the safety of home care [25]. Patient participation is enhanced by a confidential and respectful nurse-patient relationship [5, 20, 21, 26]. 

The research indicates that time, place used to counsel, and the environment can impact the quality of counselling [18]. Studies have shown that patients prefer the counselling situation to be unrushed and the atmosphere to be acknowledgeable, safe, respectful, and pleasant [27, 28]. A peaceful setting and flexible use of counselling time are relevant especially to the success of counselling for elderly patients. Also, open and honest communication between patients, family members, nursing staff, and doctors has been found to have special significance to the success of home care related counselling [29]. The consistency of counselling is enhanced by collaboration and communication between nurses, doctor and possibly a social worker providing counselling [30]. Also family members need information about the patient's illness and its treatment methods to be able to support care at home [18, 31]. 

The purpose of this study was to describe patients' and their family members' experiences about counselling in ED, and follow if these experiences possibly change after the educational intervention [15] for the whole nursing staff of the certain ED ward. This kind of research is needed for developing counselling and also getting knowledge about what kind of educational tools could be useful for educating staff working in shifts.

The study aimed (1) to describe and compare patients' experiences of content and amount of counselling and of counselling situation before and after the educational intervention; (2) to describe and compare patients' family members' experiences of content and amount of counselling and of counselling situation before and after the educational intervention.

## 2. Materials and Methods

The design was a followup, pretest-posttest study which included an intervention. The intervention consisted of online continuing education for the ED staff. On the basis of the results of the first study phase in 2003 [18], online continuing education was implemented for the whole ED staff between 2004 and 2005 [15]. The focal areas of online education were as follows: principles of high-quality counselling offered to patients and family members in ED, written after-care instructions, telephone counselling, and challenging situations (e.g., aggressive patients, critically ill patients and their family members, grieving family members, and family members in shock). These materials were presented in six sessions, which were added to the Web-based study area sequentially during six months. Discussion topics concerning these areas were available. Participants had the chance to read the material for each theme and then discuss the topic with each other in the Web discussion forum, at the time which was suitable for them. As a part of the educational intervention, written home care instructions were developed, together with the entire staff.

The article outlines the possible impact of the intervention on patients' and their family members' experiences of counselling by comparing the results of the two study phases, conducted in 2003 and 2008 [14, 15, 18]. Of course we understand that the time frame between the measures is quite long and many other factors have been present in the ED ward but still, it is interesting to see how the experiences might change between the measures. Besides, the online education and developing written home care instructions as a part of it, the staff also participated systematically in ED ward meetings which included counselling topics, held by the staff and nursing students during the years. This might have helped the staff to keep in mind and deepen the focal issues which the online education focused on.

The data were collected in both phases by using a questionnaire containing 50 questions, of which 34 were rated on a Likert scale. Those measured the content and amount of counselling and the counselling situation (see the content of the items in Tables 1–3). Others were demographic questions, and two were open-ended concerning ideas about developing counselling. The questionnaire was not modified in 2008 because the original questionnaire provided reliable information and data collected with the same questionnaire could be considered comparable [14, 18].

The sampling method used in the study was purposive random sampling because we wanted to achieve as representative a sample as possible. Patients and their family members were included in the study, if they understood the information related to the study and were willing to participate. In 2003, the entire ED staff distributed questionnaires to a total of 250 patients and in 2008 to a total of 100 patients during the morning, the evening, and the night shifts. Family members received 250 questionnaires in 2003 and 150 questionnaires in 2008.

Ethical guidelines were followed in the study by asking questions that did not offend the participants, they were just asked to describe their own experiences during the ED visit. Participation in the study was voluntary, and an appropriate research permit and ethical approval were granted by the Hospital District. The anonymity of participants was maintained throughout the study process. Participants completed the questionnaire anonymously and returned it in a sealed envelope to the nurses upon leaving the department or by post in a sealed envelope to the secretary of the department's nursing director [14, 15, 18].

The data were analyzed using SPSS for Windows version 15, by descriptive statistics, for making comparisons and for looking at the possible change after the intervention. Participants were divided into groups according to age, gender, educational background, and marital status to enable comparison and analysis of differences between individual variables using the Mann-Whitney *U* test. Further, the data were analyzed by using the waiting time, less or more than 3 hours. The median waiting time of patients was 240 min and of family members who accompanied them, 180 min. 

The return rate for the first study phase was 43% (*n* = 107) and for the latter study phase 77% (*n* = 77). In 2003, 45% (*n* = 48) of the patients were women and 55% (*n* = 58) were men. Their ages ranged from 12 to 86 years. In 2008, 54% (*n* = 42) of the patients were women and 46% were men (*n* = 35). Their ages ranged from 10 to 88 years. In 2003, 69% (*n* = 63) of the family members were women and 31% (*n* = 29) were men. Their ages ranged from 18 to 76 years. In 2008, 71% (*n* = 66) of the family members participating in the study were women and 29% (*n* = 27) were men. Their ages ranged from 17 to 80 years. 

## 3. Findings

When comparing the results from the 2003 and 2008 studies, it can be noted that patients' family members were slightly more satisfied with the counselling they received in 2008 than they were in 2003. Also patients' satisfaction was increased in most issues. In addition, patients and family members who had waited in the ED less than three hours reported increased satisfaction with information about the illness, examinations, care procedures, and waiting time. Some main trends and especially statistically significant findings are presented in the findings section. All findings which describe items concerning content and amount of counselling and the counselling situation for both patients and family members are presented in Tables 1–3. 

### 3.1. Patients' and Family Members' Experiences of the Content of Counselling

Patient satisfaction with counselling on examinations, treatments, monitoring equipment, and examination facilities had increased. There is still a need to develop medication counselling as patients had received adequate information about the medication they received but not about its effects (Table 1). In addition, patient ratings of being informed about eating, drinking, and freedom of movement during the wait had declined between measures. Also the patients' satisfaction with getting information on test results had decreased when comparing results between 2003 and 2008.

A comparison between the results of the 2003 and 2008 studies showed that family members' satisfaction with the counselling they received had slightly improved, in all measured areas. The highest increase in satisfaction (*P* = .01) with counselling was noted for factual information about whether the patient is permitted and has the chance to eat and drink during the wait in the ED. Also, family members reported greater satisfaction with counselling on information about the examinations (*P* = .05) and care procedures performed on the patient (*P* = .05) in the ED (Table 1). Those who had waited less than 3 hours had received more information on how the patient was progressing through the ED (*P* = .039), examinations (*P* = 006), examination facilities (*P* = .002), and the patient's possibility to eat (*P* = .004) and move about during the wait (*P* = .008) in 2008. They had also received more information on their chance to stay with the patient during the wait and felt that the counselling given by nurses was clearer than it was in 2003.

### 3.2. Patients' and Family Members' Experiences of the Amount of Counselling

Patients were more satisfied in 2008 than they were in 2003 with counselling on their illness, care procedures, and examination provided by the nursing staff and doctors, whereas satisfaction with counselling on post-discharge care given by the nurses had declined. Patients were less satisfied especially with counselling on medications given by both the nursing staff and doctors. Patients' experiences of the information for their family members had improved when compared with the 2003 study and also with respect to counselling on examinations, care procedures, postdischarge care, and medications. Patients were less satisfied only with counselling on their illness for the family members. In addition, the presence of a family member was considered more important in 2008; and written instructions were more appreciated (*P* = .01) in 2008. Importance of the presence of family members had increased from the viewpoint of patients between measures (Table 2). Family members' experiences had improved in all measured areas between 2003 and 2008, statistically significantly concerning examinations (*P* = .05), care procedures (*P* = .05) from nursing staff, and information given to family members about examinations (*P* = .05) (Table 2). 

### 3.3. Patients' Experiences of the Counselling Situation

Keeping the patient informed, had improved in the ED between measures with information about how their case was progressing through the ED. In 2008, they also were more satisfied with information on where one can wait than in 2003 (*P* = .05). Instead, when comparing the results concerning the place and time for counselling, it can be noted that patients' ratings of these had declined between measures (Table 3).

The experiences of the counselling situation of family members who accompanied the patients in the ED had improved slightly between measures. They were more satisfied with keeping them abreast of the situation than they were in 2003. The fact that family members perceived the counselling given by the nurses to be clearer and less cursory than they did in 2003 was also evidence of positive progress. The fact that satisfaction with information about the waiting time had increased can be regarded as a step in the right direction since it emerged as one of the critical development goals in the 2003 study. The family members reported satisfaction with being heard while communicating with the nursing staff (Table 3).

## 4. Discussion

When comparing the results of the two study phases conducted in the ED in 2003 and 2008, it can be stated that the quality and quantity of counselling received by both patients and their family members had improved slightly. This is the situation especially concerning patients' family members. As the satisfaction perceived by patients and their family members had increased, it can be thought that the counselling they received better meets their expectations and information needs than it did before. The results allow us to make the cautious assumption that the educational web-based intervention and the development of written patient instructions have had an impact on the evolution of the quality of counselling. The same kind of slightly positive results concerning educational web-based intervention are shown also by Kontio et al. [32]. However, we have to be cautious in saying that the changes are due to the online educational intervention because the time frame is quite long and also of course other changes have happened in ED. But the changes concerned especially increased satisfaction of patients' family members which was the main focus of the education.

The satisfaction of both patients and family members with counselling on the patient's illness, examinations, and care procedures had increased in 2008 when compared with the results of the 2003 study. Patients' and their family members' experiences of being kept informed had improved from 2003 to 2008. Keeping them informed is crucial point for satisfaction during waiting time shown also by Boudreaux and O'Hea [33]. More attention should still be paid for example, on councelling concerning effects of medications. This is especially important for patients discharged from hospital and for management of care at home.

There had been essential improvement in the quality of counselling for family members who had waited in the ED less than 3 hours. The satisfaction of those family members who had waited with the patient in the ED more than 3 hours remained unchanged. Previous studies have also found that a shorter waiting time is related to greater satisfaction with the content of counselling [14, 18]. This gives us advice to develop care processes on ED's.

Previous studies have reported that patient satisfaction is enhanced by a respectful nurse-patient relationship [5, 20, 21, 26, 34–36]. In this study, patient satisfaction with being heard had declined. It is especially important to pay attention to this issue. The experience of being heard is relevant also according to Boudreaux and O'Hea [33]. Patients will be better able to tolerate uncertainty and waiting if they feel that they are appreciated as a human being and that their views are being listened to. In this study, the family members that accompanied the patient reported that they had been heard when discussing with the nurses, whereas in their opinion, doctors had not paid enough attention to the information they gave about the patient. The study by Muntlin et al. 2006 [37] also showed that family members appreciate the experience of being heard and encouraged by nursing staff and doctors. 

## 5. Limitations

Reliability of a study can be evaluated on the basis of measurement, data collection, and analysis of the results [38]. The research results presented here are a description of reality from the perspective of patients and their family members. When designing the questionnaire for the initial survey, we wanted to make sure that the questionnaire was functional, logical, comprehensible, and easy to use. This was done by conducting a preliminary survey where we distributed 10 questionnaires, eight of which were returned. At that time, there was no need to modify the instrument on the basis of the feedback. 

The content validity of this study is enhanced by the fact that the concepts included in the instrument were derived from the theoretical framework for the study and adjusted to allow measurement already for the initial survey, which was described in more detail in our previous publications [14, 15, 18]. The stability and internal consistency reliability of the instrument were tested and confirmed as a result of this study [38]. Questionnaire length may affect the reliability of the measurement results. The return rate was nonetheless good, which improves the generalizability of the results. Reliability is also enhanced by the fact that the questionnaires were distributed to the companions at different times of the day over a long period of time. The time frame between baseline data collection and followup was quite long and of course many other issues than online education for the staff happened during that time. The main focus in this follow-up research was to strengthen family-centred care in ED and we say that our findings show some progress in that area. Our findings can be used to design measures intended to develop family-centred counselling practices in EDs, especially in planning further online education for staff working in shifts. 

## 6. Conclusion and Implications for Nursing

As a conclusion, it can be said that the quality of counselling provided to patients and their family members has improved as a whole, especially experiences of family members. This gives positive feedback to the education provided to the whole ED staff in web. This kind of education concerning the content of counselling can be used in developing ED care. The main focus of the education was in developing family-centred care and this aim was at least partly achieved. Special attention should still be paid to listening to the patients' and their family members' experiences and views, as the experience of being heard is important with regard to the patient's and family members' control over the situation in ED and also in terms of motivation for home care. Patients and their family members should receive more counselling on medications and their effects. From the perspective of developing family centred care, it would be necessary to provide patients and their family members with information and participate in counselling together. During the time when they wait for progressing of care in ED, could be planned more in a way which advances their coping at home as well as possible.

## Figures and Tables

**Table 1:** Patients' and family members' experiences of the content of counseling.

	Patient			Family member			
The content of counselling	2003	2008	Difference	2003	2008	Difference	*P*
	M	M		M	M		
Information on whether one is allowed to eat and drink	4.45	4.38	−0.07	3.78	4.72	**0.94**	∗∗
Information on whether one is allowed to move around	4.41	4.21	−0.20	4.18	4.63	**0.45**	
Information on examinations performed	4.69	5.03	0.34	4.31	4.89	**0.58**	∗
Information on care procedures performed	4.59	4.88	0.29	4.4	4.63	**0.23**	
Instruction on examination room locations	5.11	5.31	0.20	4.9	5.34	**0.44**	∗
Information on test results	5.08	4.93	−0.15	4.49	4.76	**0.27**	
Information on medication received	5.01	5.10	0.09	4.79	4.97	**0.18**	
Information on effects of medications	4.76	4.58	−0.18	4.28	4.44	**0.16**	
Information on why various procedures are performed	4.77	4.71	−0.06	4.67	4.68	**0.01**	
Information on patient monitoring equipment	4.57	4.72	0.15	4.02	4.72	**0.70**	
Information on how to keep in touch with the outside world	4.11	3.90	−0.21	3.61	4.09	**0.48**	
I knew about the transfer to a ward or elsewhere	4.74	4.65	−0.09	4.76	5.08	**0.32**	
Relevance of instructions to participation in care	5.03	4.93	−0.10	4.65	4.78	**0.13**	
Clear instructions on which facility to contact	4.71	5.04	0.33	4.44	4.70	**0.26**	

* ≤ 0.05, ** ≤ 0.01, *** ≤ 0.001.

Mann-Whitney *U* test.

**Table 2:** Patients' and family members' experiences of the amount of counseling.

	Patient			Family member			
Amount of counselling	2003	2008	Difference	2003	2008	Difference	*P*
	M	M		M	M		
Information from nursing staff							
On illness-related issues	5.02	5.15	0.13	4.84	4.99	0.15	
On examinations performed	5.16	5.19	0.03	4.74	5.19	0.45	∗
On care procedures performed	5.26	5.29	0.03	4.81	5.17	0.36	∗
On post-discharge care related to illness	4.92	4.90	−0.02	4.88	4.93	0.05	
On medications received	5.06	4.81	−0.25	4.73	5.16	0.43	

Information from doctor							
On illness-related issues	5.08	5.13	0.05	4.56	5.05	0.49	
On examinations performed	5.15	5.15	0.00	4.66	5.02	0.36	
On care procedures performed	5.15	5.25	0.10	4.67	5.01	0.34	
On post-discharge care related to illness	4.86	4.99	0.13	4.59	4.90	0.31	
On medications received	5.10	4.83	−0.27	4.59	4.88	0.29	

Experience on the information given to the family member							
On illness-related issues	4.78	4.67	−0.11	5.04	5.17	0.13	
On examinations performed	4.62	4.74	0.12	5.00	5.27	0.27	
On care procedures performed	4.55	4.93	0.38	5.00	5.33	0.33	∗
On post-discharge care related to illness	4.49	4.79	0.30	4.86	5.04	0.18	
On medications received	4.46	4.92	0.46	4.91	5.18	0.27	

Experience of instructions given							
Importance of the presence of family members	4.93	5.11	0.18	5.60	5.61	0.01	
Confidence in coping at home with the help of instructions	5.20	5.08	−0.12	4.99	5.38	0.39	
I received information only when I asked for it	2.94	3.04	0.10	3.32	3.16	−0.16	
Written instructions necessary for coping at home	4.16	4.88	0.72	4.69	5.19	0.50	∗∗
Comprehensibility of instructions	5.24	5.33	0.09	5.37	5.41	0.04	

* ≤ 0.05, ** ≤ 0.01, *** ≤ 0.001.

Mann-Whitney *U* test.

**Table 3:** Patients' and family members' experiences of the counseling situation in the emergency department.

	Patient			Family member			
Counseling situation	2003	2008	Difference	2003	2008	Difference	*P*
	M	M		M	M		
Information on where one can wait	5.26	5.52	0.26	5.24	5.45	0.21	∗
Estimate of waiting time	4.06	3.92	−0.14	3.55	3.81	0.26	
Information on how the patient was progressing through the department	4.26	4.54	0.28	4.33	4.64	0.31	
Clarity of information from the doctor	5.10	5.14	0.04	4.54	4.78	0.24	
Experience of being heard by the doctor	4.99	4.99	0.00	4.8	4.70	−0.10	
Experience of being heared by the nurse	5.05	4.98	−0.07	5	5.25	0.25	
Clarity of instructions and advice	5.11	5.12	0.01	5.13	5.27	0.14	
Peacefulness of counseling space	5.08	4.97	−0.11	4.95	5.05	0.10	
Unrushed pace of counseling	5.02	4.88	−0.14	5.07	5.16	0.09	
Enough time for discussing the situation	4,83	4,84	0.01	4.77	4.99	0.22	

* ≤ 0.05, ** ≤ 0.01, *** ≤ 0.001.

Mann-Whitney *U* test.
